# Evaluation of a Rapid Immunoassay for Molecular Subphenotype Classification in Pediatric Acute Cardiorespiratory Failure

**DOI:** 10.21203/rs.3.rs-7596498/v1

**Published:** 2025-09-23

**Authors:** Colin J. Sallee, Clove S. Taylor, Matt S. Zinter, Daniela Markovic, Michelle J. Lim, Ana Costa-Monteiro, Ruth Cortado, Andreas Schwingshackl, Michael Agus, Michael Matthay, Anil Sapru

**Affiliations:** University of California Los Angeles; University of California Los Angeles; University of California San Francisco; University of California Los Angeles; University of California Davis; University of California Los Angeles; University of California Los Angeles; University of California Los Angeles; Boston Children’s Hospital, Harvard Medical School; University of California San Francisco; UCLA Pediatric Critical Care

**Keywords:** Molecular Subphenotyping, Precision Medicine, Rapid Immunoassay, Biological Heterogeneity, Clinical Trials

## Abstract

Molecular subphenotypes, identified through biomarker profiling independent of clinical diagnosis, have the potential to guide targeted therapeutics in critical care. We have previously published that intensive insulin management has subphenotype-specific beneficial effects among children with hyperglycemia accompanying cardiorespiratory failure. However, due to the operational aspects of biomarker assays, prospective real-time application of subphenotype-based strategies remains daunting. This study compared biomarker values measured via a rapid immunoassay requiring minimal handling to a conventional laboratory-based multiplex assay, assessed its ability to classify subphenotypes with a parsimonious classifier, and compared clinical outcomes between rapid immunoassay-based subphenotypes. This retrospective multicenter study included re-assaying plasma samples from 269 children with acute cardiorespiratory failure and hyperglycemia. Latent class analysis (LCA) was previously used to derive hyper-inflammatory and hypo-inflammatory classes. A parsimonious classifier was fit to LCA-derived subphenotypes and produced a model consisting of IL-6, IL-8, and sTNFR-1. We found that, despite the rapid immunoassay systematically overestimating biomarker values relative to the conventional assay, biomarkers were strongly correlated between platforms (Pearson *r* = 0.87–0.93). Using the parsimonious classifier, subphenotype classifications matched between platforms in 95% of patients (n = 256/269). When compared to previously derived LCA-derived subphenotypes, rapid immunoassay-based subphenotypes demonstrated an AUC of 0.90 (95% CI 0.85–0.95). The rapid immunoassay-based hyper-inflammatory class was associated with higher mortality (26% vs. 11%; P = 0.01) and heterogeneity of treatment effect to intensive insulin management (interaction P = 0.01). Our findings suggest that subphenotyping using a rapid immunoassay is feasible and accurate, laying the foundation for future precision medicine strategies in pediatric critical care.

## INTRODUCTION

Biological heterogeneity within clinically defined patient groups is postulated to limit the success of therapeutic trials in critical care.^1^ Although patients may share the same diagnosis, their underlying pathophysiology can differ, leading to varied responses to the same intervention. Molecular subphenotypes, derived using latent class analysis (LCA) and characterized by differential elevations in inflammatory and endothelial biomarkers, have been associated with distinct clinical outcomes and heterogeneity of treatment effect (HTE).^2^ These findings, consistently observed across various critical illnesses, support the advancement of biomarker-guided precision medicine and more targeted therapeutic strategies.^3–7^

Although implementation remains challenging, discrimination of molecular subphenotypes using parsimonious classifiers, such as in Sinha et. al.,^8^ lend feasibility to bedside application; though, evaluation of this approach using biomarkers measured by a rapid immunoassay have been limited.

To investigate the performance of molecular subphenotyping strategies using a rapid immunoassay, we measured and compared biomarkers from a pediatric acute cardiorespiratory failure cohort (CAF-PINT) with previously derived LCA subphenotypes. Accordingly, the study had three objectives: (1) compare a rapid immunoassay to a conventional laboratory-based multiplex assay; (2) using a parsimonious classifier, evaluate the discriminative performance of this rapid immunoassay for subphenotype classification; (3) examine the associations between the rapid immunoassay-based subphenotypes and clinical outcomes.

## METHODS

### Study Population and Design:

This retrospective cohort study is based on the CAF-PINT study,^9,10^ in which pediatric patients with acute cardiorespiratory failure and hyperglycemia were randomized to insulin infusion targets of either 80–110 mg/dL (TG_80− 110_) or 150–180 mg/dL (TG_150− 180_). In the CAF-PINT study, 13 biomarkers reflective of inflammation and endothelial activation were assayed using the Human Magnetic Luminex^®^ Multiplex Discovery Assay (Bio-Techne, Minneapolis, MN). Using biomarker data generated from the Luminex/Multiplex assay, LCA was performed using approaches detailed by Calfee^2^ and Sinha et al.^8^ to derive hyper-inflammatory and hypo-inflammatory classes. The hyper-inflammatory class was previously shown to be associated with higher mortality and HTE from intensive insulin therapy (TG_80− 110_).^9,10^

### Ella Rapid Immunoassay Platform:

In the present study, stored plasma samples were run in triplicates on the Ella^™^ platform (Bio-Techne, Minneapolis, MN). This microfluidic-based platform automates multi-cartridge enzyme-linked immunoassays (ELISA) in under 90 minutes without manual intervention using minimal plasma volume (~ 35 μL).^11,12^ This rapid immunoassay included assays for interleukin (IL)-6 and − 8 and soluble tumor necrosis factor receptor 1 (sTNFR-1); selection of biomarkers was informed by the parsimonious classifier described below. Average intra-assay coefficients of variation (CV) were < 5% for all analytes. Measurements of analytes were performed on plasma samples across 9 plates, with batch effects minimized by using a single operator and incorporating internal standards; corresponding inter-assay CVs were < 20% for IL-6 and < 15% for IL-8 and sTNFR-1.

### Statistical Methods:

A parsimonious classifier, derived using least angle regression^13^ and fit to LCA-derived subphenotypes, produced a three-biomarker classifier consisting of IL-6, IL-8, and sTNFR-1 with the following equation:

Logit(P)=-18.146+1.854×log10(IL-6)+1.084×log10(IL-8)+3.485×log10(sTNFR-1)

The parsimonious classifier yielded an area under the receiver operating characteristic curve (AUC) of 0.94 (95% confidence interval [CI] 0.91–0.97) when evaluated against the LCA-derived subphenotypes reported in the original CAF-PINT study.

Pearson correlation coefficients (*r*) assessed the strength of association between biomarker concentrations measured via Ella and Luminex/Multiplex platforms. Deming regression was used to identify systematic biases between platforms as this method accounts for measurement errors in both explanatory and response variables.^14^

A 2×2 confusion matrix compared Ella and Luminex/Multiplex-based subphenotype assignment using the parsimonious classifier. Hyper-inflammatory class membership was assigned using the optimized Youden index.^15^ Classification agreement between assay platforms was assessed using the Kappa statistic.

The performance of Ella and Luminex/Multiplex-based parsimonious subphenotypes were compared against reference standard LCA. Discriminative performance was evaluated via AUC with 95% CIs calculated via the DeLong method.^16^ Given the low prevalence of the LCA-derived hyperinflammatory class (~ 15%), precision-recall curves were also used to test model accuracy.

## RESULTS

**Figure 1:**
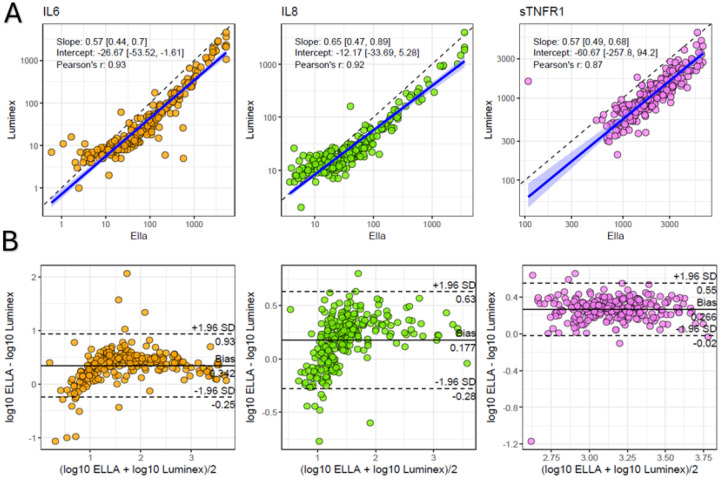
**A**. Scatterplots of IL6, IL8, and sTNFR-1 measured by Ella (x-axis) and Luminex/Multiplex (y-axis). Biomarker concentrations are in pg/mL. Deming regression lines (in blue) and Pearson correlations (*r*) are presented for each biomarker. Bootstrapping with 1000 resamples was performed to obtain robust confidence intervals for regression coefficients. Dashed lines correspond to the identity line (x=y) for each biomarker. **B**. Agreement between the Ella and Luminex/Multiplex platforms assessed via Bland-Altman plots. For each biomarker, the difference between platforms (y-axis) is plotted against the platform average (x-axis). The solid line represents the mean bias, while the dashed lines indicate the 95% limits of agreement. *Note: sTNR-1 measurements exceeding the Ella ULoQ (n=16/269, 6%) were not included in these Figures. IL6 = interleukin-6, IL8 = interleukin-8, sTNFR1 = soluble tumor necrosis factor 1.

**Figure 2:**
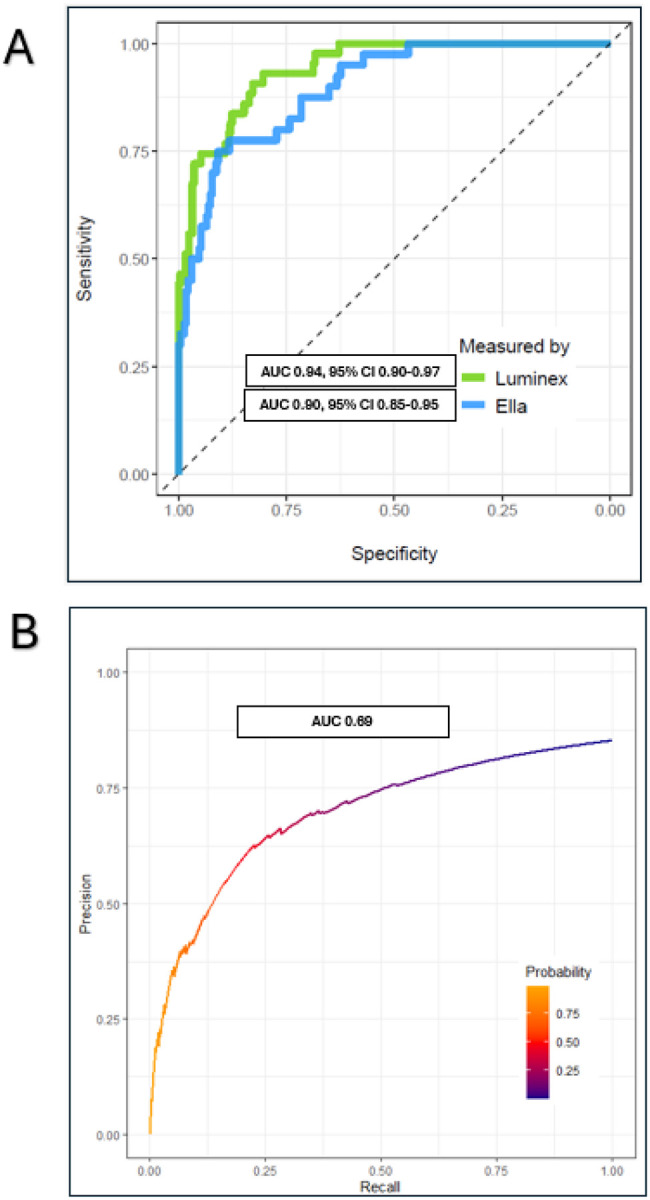
**A**. Area under the receiver operating curves (AUC) applying the parsimonious classifier to Ella (blue) and Multiplex/Luminex (green) values. Discriminatory performance was evaluated against LCA-derived subphenotypes as the reference standard. **B**. Precision-recall curve (PRC) plotting precision (positive predictive value, y-axis) against recall (sensitivity, x-axis) The PRC curve demonstrated an AUC of 0.69 against a prevalence of 0.15.

1. Analytical Comparison Between Ella and Luminex/Multiplex Platforms:
  Concentrations (in pg/ml) of IL-6, IL-8, and sTNFR1 measured across platforms showed strong positive correlations (Pearson *r* = 0.87–0.93, all P < 0.0001). IL-6 median levels were 57 (interquartile range [IQR] 18–223) on Ella versus 20 (IQR 9–80) on the Luminex/Multiplex platform. IL-8 levels were 31 (IQR 16–64) compared to 20 (IQR 13–31), and sTNFR1 levels were 2038 (IQR 1350–3182) versus 1075 (IQR 743–1652), respectively. Deming regression demonstrated proportional biases across platforms, with Ella consistently reporting higher biomarker concentrations relative to the Luminex/Multiplex platform (Fig. 1A). Bland-Altman plots further illustrate inter-assay differences (Fig. 1B).
2. Molecular Subphenotype Discrimination by Ella Using a Parsimonious Classifier:
  Subphenotype assignments generated by the parsimonious classifier using Ella values showed strong agreement with assignments generated applying the same classifier to Luminex/Multiplex values. Using a Youden index of 0.76, classifications matched in 95% of patients (n = 256/269), with a hyper-inflammatory class false-positive rate of 5.5% (Table 1). The Kappa statistic was 0.81, indicating good overall agreement between assay platforms. Figure 2 displays the discriminative performance of Ella and Luminex/Multiplex-based parsimonious subphenotypes evaluated against LCA-derived subphenotypes.
3. Clinical Associations and HTE to TG_80 − 110_ vs. TG_150 − 180_ :
  The Ella-based subphenotypes described above were compared for their class-specific outcomes. The Ella-based hyper-inflammatory class had fewer 28-day ventilator-free days (median 11.0 [IQR 0.0–21.0] vs. 22.0 [IQR 11.0–25.0]; Wilcoxon P < 0.001) and higher 90-day mortality (26% vs. 11%; Chi-square P = 0.01) compared to the Ella-based hypo-inflammatory class. Among the hyper-inflammatory class, tight glycemic control (TG_80 − 110_) significantly improved 90-day mortality (11%, n = 2/18 for TG_80 − 110_ vs. 36%, n = 10/28 for TG_150 − 180_, interaction P = 0.01).

## DISCUSSION

In this multicenter study of 269 children with acute cardiorespiratory failure, we show that IL-6, IL-8, and sTNFR-1 levels measured with a rapid immunoassay correlate with levels obtained using a conventional laboratory-based multiplex assay. When incorporated into a simple classifier, this rapid immunoassay accurately classifies LCA-derived molecular subphenotypes. Moreover, subphenotypes defined using this rapid immunoassay not only associate with clinical outcomes but also demonstrate HTE in response to intensive insulin therapy.

The partitioning of patients into clinically meaningful, biology-based subphenotypes has motivated a paradigm shift in critical care medicine. Rather than a “one size fits all” approach to patient care, recent efforts have focused on leveraging these subphenotypes to guide prognostic and predictive enrichment in clinical trials.^17^ As such, rapid diagnostic assays paired with simple classifiers could bring clinicians closer to timely and actionable data that enable precision medicine strategies at the bedside.^7^ We present an exciting first step toward near real-time subphenotype classification using a rapid immunoassay platform. We further show the rapid immunoassay, relative to conventional laboratory-based multiplex assay, minimizes manual handling, processing time, and sample volume, making it particularly attractive for pediatric applications and potential transition to point-of-care use.^18^

We observed that the Ella rapid immunoassay platform systematically overestimated biomarker values relative to the conventional Luminex/Multiplex platform. This likely drove the minor discordance between platforms, as applying Ella-based biomarker values to the parsimonious classifier produced hyper-inflammatory class false positives. Nevertheless, in the absence of a definitive gold-standard, discrepancies between platforms are to be expected.^19,20^ Deming regression coefficients can be used to mitigate inter-platform biases by linear transformation of raw biomarker values before incorporating them into the classifier. Accordingly, cross-platform validation across multiple cohorts, with varying prevalence of inflammatory states, is required prior to routine clinical implementation at the bedside.

In conclusion, our findings demonstrate that rapid immunoassays are feasible, accurate, and potentially, clinically accessible tools for near real-time identification of molecular subphenotypes in critically ill children. This work represents an initial step toward enabling prospective rapid subphenotyping for enrichment in clinical trials, with the goal of delivering the right treatment to the right child at the right time.

## Figures and Tables

**Table 1:** 2×2 confusion matrix of molecular subphenotype assignment using the three-variable (IL-6, IL-8, sTNFR-1) classifier applied to Ella and Luminex/Multiplex biomarker data. Youden index probability cut-off of 0.76 was used to assign hyper-inflammatory class.

	Luminex/Multiplex Hypo-inflammatory	Luminex/Multiplex Hyper-inflammatory	Total
Ella Hypo-inflammatory	223	0	223
Ella Hyper-inflammatory	13	33	46
Total	236	33	269

## Data Availability

The datasets used and/or analyzed during the current study are available from the corresponding author on reasonable request.

## Abbreviations

LCA: latent class analysis
HTE: heterogeneity of treatment effect
ELISA: enzyme linked immunosorbent assay
AUC: area under the receiver operating characteristic curve
IL: interleukin
sTNFR-1: soluble tumor necrosis factor receptor 1
